# Facultative amphidromy and pelagic larval duration plasticity of *Rhinogobius formosanus* (Teleostei, Gobioidei)

**DOI:** 10.3897/zookeys.951.50429

**Published:** 2020-07-22

**Authors:** Te-Yu Liao, Wen-Chien Huang, Yoshiyuki Iizuka, Ming-Tai Chou, Jen-Chieh Shiao

**Affiliations:** 1 Department of Oceanography, National Sun Yat-sen University, Kaohsiung 804, Taiwan; 2 Doctoral Degree Program in Marine Biotechnology, National Sun Yat-sen University, Kaohsiung 804, Taiwan; 3 Doctoral Degree Program in Marine Biotechnology, Academia Sinica, Taipei 115, Taiwan; 4 Institute of Earth Sciences, Academia Sinica, Taipei 115, Taiwan; 5 Printech Electronics Corporation, New Taipei 235, Taiwan; 6 Institute of Oceanography, National Taiwan University, Taipei 106, Taiwan

**Keywords:** *COI* sequences, diadromous, goby, landlocked, otolith

## Abstract

*Rhinogobius formosanus* Oshima, 1919 has long been considered an amphidromous goby. However, a landlocked population recently found in the Jingualiao Creek upstream of the Feitsui Reservoir in Taipei suggests that *R. formosanus* may complete its life in the river. This study aims to verify the habitat use of the landlocked population of *R. formosanus* collected from the Feitsui Reservoir and an amphidromous population collected in Malian Creek using otolith Sr:Ca ratio analysis. The hypothesis that early life history varies between the landlocked and migratory gobies was also tested. Genetic analyses show that the Feitsui Reservoir and Malian Creek populations are not genetically different. *Rhinogobius formosanus* from Malian Creek showed high-to-low otolith Sr:Ca ratios suggesting that these specimens spent a planktonic larval stage in the sea followed by a freshwater life at later stages. In contrast, *R. formosanus* from the Feitsui Reservoir showed constant lower otolith Sr:Ca ratios, implying a landlocked life history of fish in the creek upstream of the reservoir. In addition, the analysis of growth increments showed a longer pelagic larval duration for the fish in the Malian Creek (58.8 days) than those in the Feitsui Reservoir (38.8). Variation of pelagic larval duration in two genetically homogenous populations implies acclimatization to the reservoir by the landlocked gobies. This study shows that *R. formosanus*, like some other congeners, is capable of adapting to a freshwater landlocked environment in its early developmental stage and supports the hypothesis that landlocked populations may have a shorter pelagic larval duration.

## Introduction

Amphidromy is a diadromous behavior that applies to larvae living in the estuary or sea followed by the post-larvae return to a river where the fish are hatched (McDowall 2007). Amphidromous fishes are more common in the tropics and their planktonic larvae in the sea may facilitate distant dispersal (Lester and Ruttenberg 2005). Amphidromous gobies showed diverse life history traits, distribution ranges and genetic structure among populations. *Sicyopterus japonicus* Tanaka, 1909 and *S. lagocephalus* Pallas, 1770, for example, are two small amphidromous gobies with a long pelagic larval duration (PLD) of 133 to 266 days and distributed across ranges of c. 2400 and 18000 km, respectively (Shen and Tzeng 2002; Hoareau et al. 2007; McDowall 2007). The dispersal of the former is documented by genetic homogeneity across the distribution range (Watanabe et al. 2006; Ju et al. 2013), but the later shows high population structure across the Indo-Pacific Barrier (Lord et al. 2012). Narrowly endemic species, such as *Sicyopterus aiensis* Keith, Watson & Marquet, 2004 and *S. sarasini* Weber & de Beaufort, 1915 have relatively shorter PLD’s of c. 80 days and do not display genetic structure across their distribution areas in Vanuatu and New Caledonia, respectively (Lord et al. 2010, 2012).

The reconstruction of ontogenetic life stages of fish at different habitats usually relies on the analysis of otolith microstructure and chemical compositions. Fish otolith is a biomineralized structure that accretes with time by adding a growth increment on the surface (Campana and Neilson 1985; Rogers et al. 2019). Counting otolith daily growth increments can reconstruct ontogenetic stages, such as PLD and demersal life stage. In addition, otolith strontium:calcium (Sr:Ca) ratios are extensively applied to study the migration between the sea and rivers for various fishes (Tzeng et al. 2002; Shiao et al. 2016; Lozys et al. 2017; Tran et al. 2019). This is because the higher Sr concentration in sea water than in fresh water allows marine fish to deposit relatively higher Sr contents in the otolith (Campana 1999; Brown and Severin 2009).

**Figure 1.**
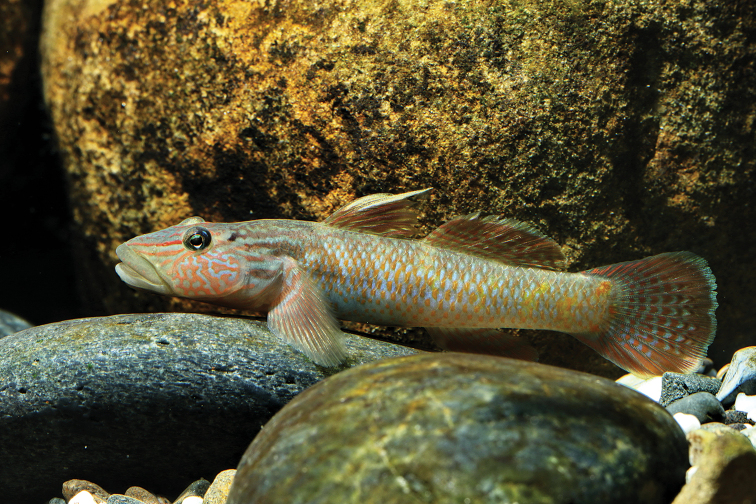
*Rhinogobius formosanus*. Male; approximately 7 cm TL, specimen not preserved.

The genus *Rhinogobius* Gill, 1859 is a group of small fishes distributed in East Asia. Species of this genus are splendid and colorful and becoming popular in the aquarium trade. Various life histories are observed in *Rhinogobius*, including amphidromous and landlocked forms (Tsunagawa and Arai 2008; Shiao et al. 2015; Yamasaki et al. 2015), while some species, such as *R. candidianus* (Regan, 1908) and three undescribed species from Japan were considered facultatively amphidromous, and either migratory or landlocked depending on whether passage to the sea is possible (Tsunagawa and Arai 2008). *Rhinogobius formosanus* Oshima, 1919 (Fig. 1) is a colorful goby easily distinguished from its syntopic congeners by numerous irregular stripes on the cheek. This species is sexually dimorphic with an extended first dorsal fin, longer snout, and intensive coloration in adult males while gravid females have a bluish abdomen (Chen and Fang 1999). *Rhinogobius formosanus* is distributed in northern Taiwan and Fujian, China (Chen and Fang 1999; Yuan et al. 2012) and has long been considered an amphidromous fish (Chen and Fang 1999), inhabiting running water close to the tidal reach of small tributaries and creeks directly connected to the sea. Chen and Fang (1999) stated that *R. formosanus* could be landlocked, but detailed information was not provided. Recently, a population of *R. formosanus* was found upstream of the Feitsui Reservoir (FR) in Taipei. This population may be landlocked, since the dam of 122.5 m height completely blocks upstream migration of aquatic life. Even if the fish larvae survive the downstream passage from the reservoir, the juvenile and adult fish cannot return upstream of the dam (Chang et al. 1999). The Feitsui Reservoir was built at the upper reaches of Tamsui River in 1987, so the FR population has probably been landlocked since then if this species was native to that area, or afterwards if it was artificially released.

Based on the above facts, this study aims to test two hypotheses. First, *R. formosanus* upstream of FR reside in the river for their whole life while conspecifics not blocked by dams are amphidromous. Secondly, we hypothesize that the landlocked goby will have a shorter larval planktonic stage than amphidromous conspecifics since the later may spend more time drifting to the sea during the early larval stage, dispersing away from the coasts, then returning to the estuary at the post-larval stage. To test the hypotheses, the early life history of the fish was reconstructed by reading daily growth increments and analyzing otolith Sr:Ca ratios. In addition, mitochondrial cytochrome oxidase subunit I (*COI*) fragments were sequenced to provide molecular data of genetic differentiation between landlocked and amphidromous populations in order to infer the landlocked life history, if any, as a consequence of acclimation or adaptation.

## Material and methods

### Samplings

**Figure 2.**
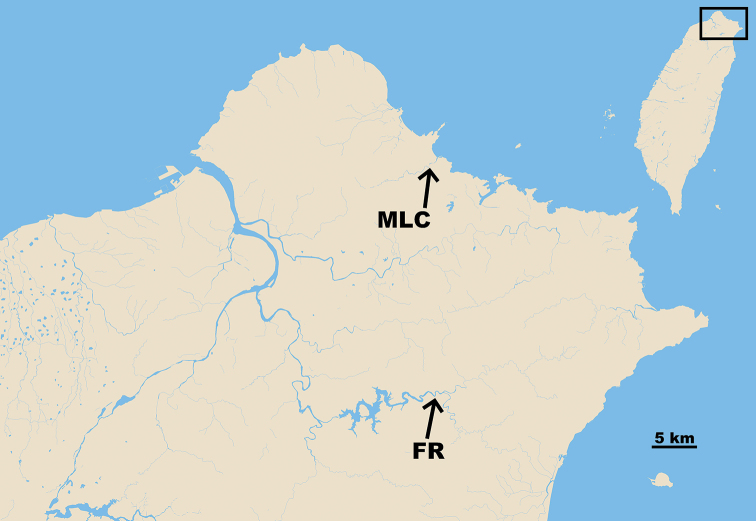
Sampling localities of *Rhinogobius formosanus* in Taiwan. Location codes are given in Table 1. FR, Feitsui Reservoir (GPS: 24°55'46.8"N, 121°41'29.6"E), representing a landlocked population; MLC, Malian Creek (GPS: 25°10'17.2"N, 121°40'47.7"E), representing an amphidromous population.

A total of 20 specimens of *R. formosanus* were collected from two creeks in northern Taiwan:Jingualiao Creek, which flows into the upstream area of FR, representing a landlocked population with syntopic congeners *R. candidianus* and *R. similis* Gill, 1859; and Malian Creek (MLC), representing an amphidromous population with syntopic congener *R. similis*, directly connected to the sea (Fig. 2; Table 1). The FR dam is approximately 50 km away from the Tamsui River mouth and the sampling site at the Jingualiao Creek was approximately 20 km upstream of the reservoir dam. Sampling sites for MLC were approximately 1 km away from stagnant water and 2 km away from the river mouth while sampling site for FR was 10–20 m away from the lentic reservoir. Both sampling sites were lotic. All specimens were collected using a hand net and anesthetized immediately after capture. They were brought back to the lab for further molecular and otolith analyses. All specimens were preserved in 95% ethanol, cataloged and deposited in the collection of the Department of Oceanography, National Sun Yat-sen University (DOS), Kaohsiung. The voucher numbers of specimens are as follow: FR, DOS 03534–2, –11, –14, –15, –16, –19, –33, –35, –36, –37; MLC, DOS 02416–1, –2, –5, –6, –7, –12, –13, –15, –20, –22. All the procedures in this study were approved by the “Institutional Animal Care and Use Committee of National Taiwan University”.

### Molecular analyses

DNA was extracted from fin clips using GeneMark DNA Purification Kit (GMbiolab, Taichung, Taiwan). The mitochondrial *COI* gene was amplified by polymerase chain reaction (PCR) with universal primers designated by Ward et al (2005): FishF1 (5’-TCAACCAACCACAAAGACATTGGCAC-3’) and FishR1 (5’- TAGACTTCTGGGTGGCCAAAGAATCA-3’). The total reaction volume of the PCR was 25 μL, containing 1 μL of template DNA (50–200 ng μL^-1^), 3 μL of 10× buffer, 2 μL of dNTPs (2.5 mM), 1.2 μL of each primer (10 μM), 0.13 μL of ProTaq Plus polymerase (Protech, Taipei) and 16.47 μL of deionized water. PCR cycling conditions included an initial denaturation at 94 °C for 5 min, followed by 36 cycles of denaturing at 94 °C for 30 sec, annealing at 50 °C for 30 sec, and extension at 70 °C for 1 min, and a final extension at 72 °C for 8 min. After checking qualities by electrophoresis, PCR products were purified using the SAP-Exo purification kit (Jena Bioscience, Jena) according to the manufacturer’s protocols. Sequencing was conducted by an ABI 3730 automated sequencer. Newly generated sequences were edited manually using MEGA version X (Kumar et al. 2018) and translated into amino acids to ensure absence of insertions, deletions, or stop codons. All sequences used in this study were submitted to the GenBank online database (Accession numbers MN187015–MN187034).

The genetic diversity indexes of haplotype diversity (h) and nucleotide diversity (π) were calculated in DnaSP version 6 (Rozas et al. 2017). A minimum spanning network of haplotypes was reconstructed by PopART version 1.7 (Leigh and Bryant 2015) to infer the interrelationships among the haplotypes.

### Measurement of otolith Sr:Ca ratios and growth increments

Sagittal otoliths were extracted from eight and five specimens from the FR and MLC populations, respectively. The otoliths were cleaned and embedded in Epofix resin (Struers, Denmark) before repeated grinding and polishing along the sagittal plane until the core was revealed on the surface. The otoliths were coated with a layer of carbon (Q150TE, Quorum Technologies Ltd., UK) to increase the electron conductance when the otoliths were analyzed by the electron probe microanalyzer (EPMA, JEOL JXA-8900R, JEOL, Japan). Quantitative analyses of Sr and Ca were conducted along a transect from the otolith core to the edge at 10 μm intervals. Electron beam conditions were 15 kV for the acceleration voltage and 3 nA for the current, with a 5 × 4 μm rectangular scanning beam size. The wavelength dispersive spectrum at the Sr Lα peak position was measured for 80 s and each of the upper and lower baselines for 20 s. The peak concentration of Ca Kα was measured for 20 s and each of the upper and lower baselines for 10 s. Synthesized strontianite [(Sr_0.95_Ca_0.05_) CO_3_; NMNH R10065] and aragonite (CaCO_3_) were used as standards to calibrate the concentration of Ca and Sr, respectively, in the otoliths. The Sr:Ca ratios were calculated after a correction using the PRZ (phi‐rho‐z) method (Goldstein et al. 1992). The detection limits were better than 500 ppm for Ca and Sr and the analytical errors were smaller than 0.05 wt% in Sr (Iizuka 2012). The otolith Sr:Ca ratios < 4 × 10^-3^ and > 5 × 10^-3^ were regarded as freshwater and marine residences, respectively. The values between 4 × 10^-3^ and 5 × 10^-3^ represent the transition between river and marine habitats.

After the analysis of otolith Sr:Ca ratios, the otoliths were polished to remove the carbon coating and etched with 0.1 M HCl for 10–15 s to enhance the contrast of growth increment observed under a compound light microscope (Olympus BX 51, Japan). Two experienced researchers counted the otolith growth increments from the core to a high‐contrast growth increment (an otolith check), or to a structural transition from clear concentric rings to ambiguous growth increments. This otolith check, appearing at the transition of high-to-low otolith Sr:Ca ratios, represented the ontogenetic change from pelagic larvae to demersal juvenile living in the river. If the two counts differed, the otolith was examined once more and final age was determined after discussion. The maximal distance from the core to the otolith check, or to a structural transition was also measured, which was further divided by the number of the growth increments to estimate the mean otolith growth rate during the pelagic larval stage of the gobies. One-way ANOVA was used to compare the otolith Sr:Ca ratios representing marine and freshwater life stages. The student’s t-test was used to compare the PLD and otolith growth increment width between the landlocked and amphidromous gobies. Statistical significance was set at α = 0.05.

## Results

### Molecular analyses

A fragment of mtDNA *COI* (555 bp) from 20 specimens obtained from two localities (Fig. 2) was analyzed. In total, six haplotypes were identified with two collected in FR and five in MLC. Among the six haplotypes, only one was shared by both populations and the rest were unique to either population. Both haplotype (π) and nucleotide (*h*) diversities of FR were lower than those of MLC (mean ± SD; h: 0.200 ± 0.154 and π: 0.00036 ± 0.00028 vs. h: 0.800 ± 0.100 and π: 0.00244 ± 0.00064). Total haplotype and nucleotide diversity were 0.574 ± 0.122 and 0.00161 ± 0.00046, respectively (Table 1).

**Table 1.** Sampling locations and diversity indices of *COI* fragment of *Rhinogobius formosanus*. *N* for sample size of molecular analyses; *n* for sample size of otolith study; *h* for haplotype diversity; π for nucleotide diversity.

Locality	Abb.	*N*	*n*	COI sequence/ haplotypes	*h* ± SD	*π* ± SD
Feitsui Reservoir	FR	10	8	10/2	0.200 ± 0.154	0.00036 ± 0.00028
Malian Creek	MLC	10	5	10/5	0.800 ± 0.100	0.00244 ± 0.00064
	Total	20	13	20/6	0.574 ± 0.122	0.00161 ± 0.00046

**Figure 3.**
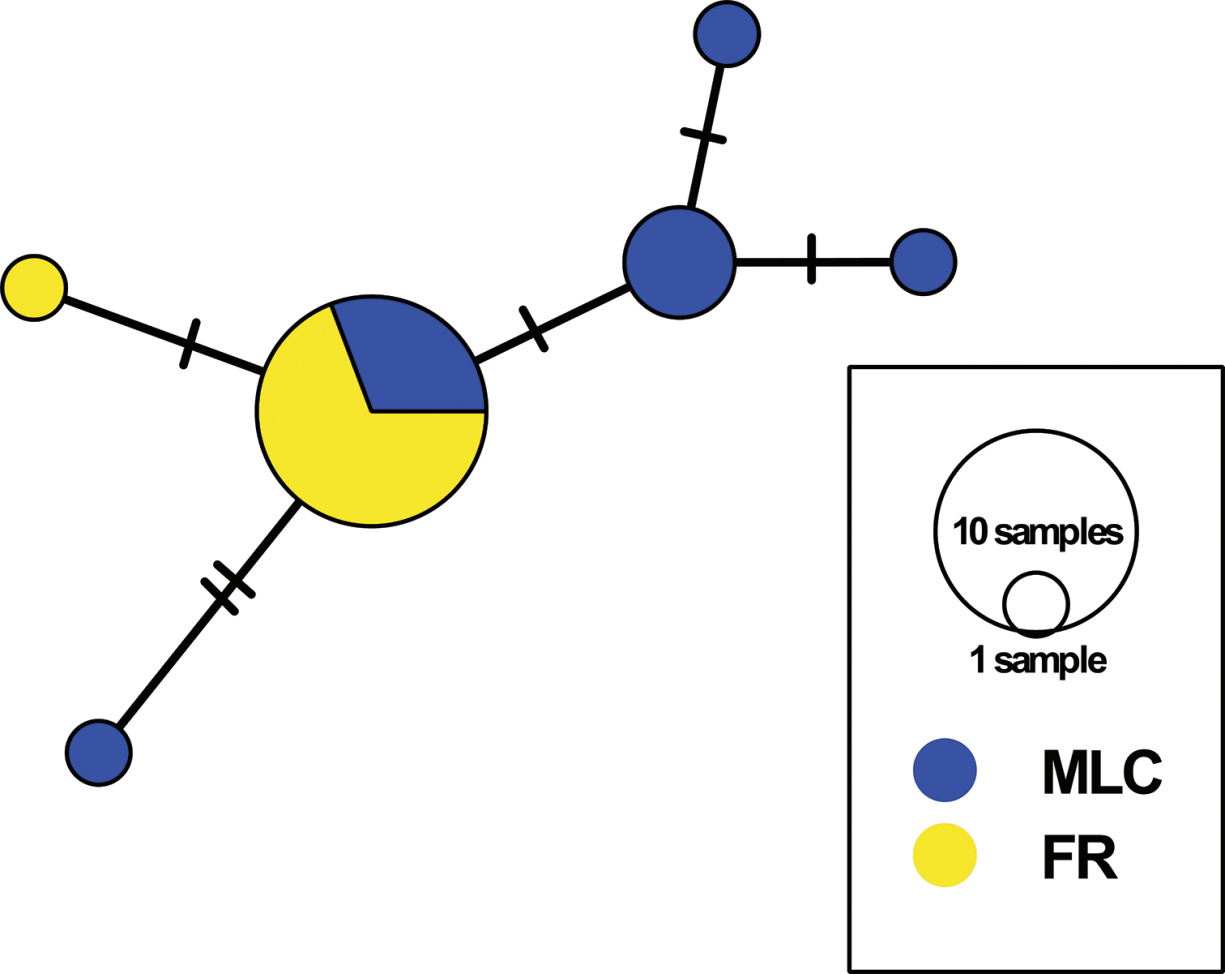
Minimum spanning network built from 20 *COI* sequences of *Rhinogobius formosanus* with six haplotypes. Colors represent correspondent sampling sites; pie chart sizes are proportional to the number of individuals; short bars represent haplotypes not collected in this study. FR, Feitsui Reservoir, representing a landlocked population; MLC, Malian Creek, representing an amphidromous population.

The haplotype network showed that all *COI* sequences obtained from the two populations were mixed. Monophyly of either population was not recovered, with the shared haplotype comprising 13 individuals and the rest of the five haplotypes each consisting of not more than three fish (Fig. 3).

### Otolith Sr:Ca ratios and pelagic larval durations of the gobies

**Figure 4.**
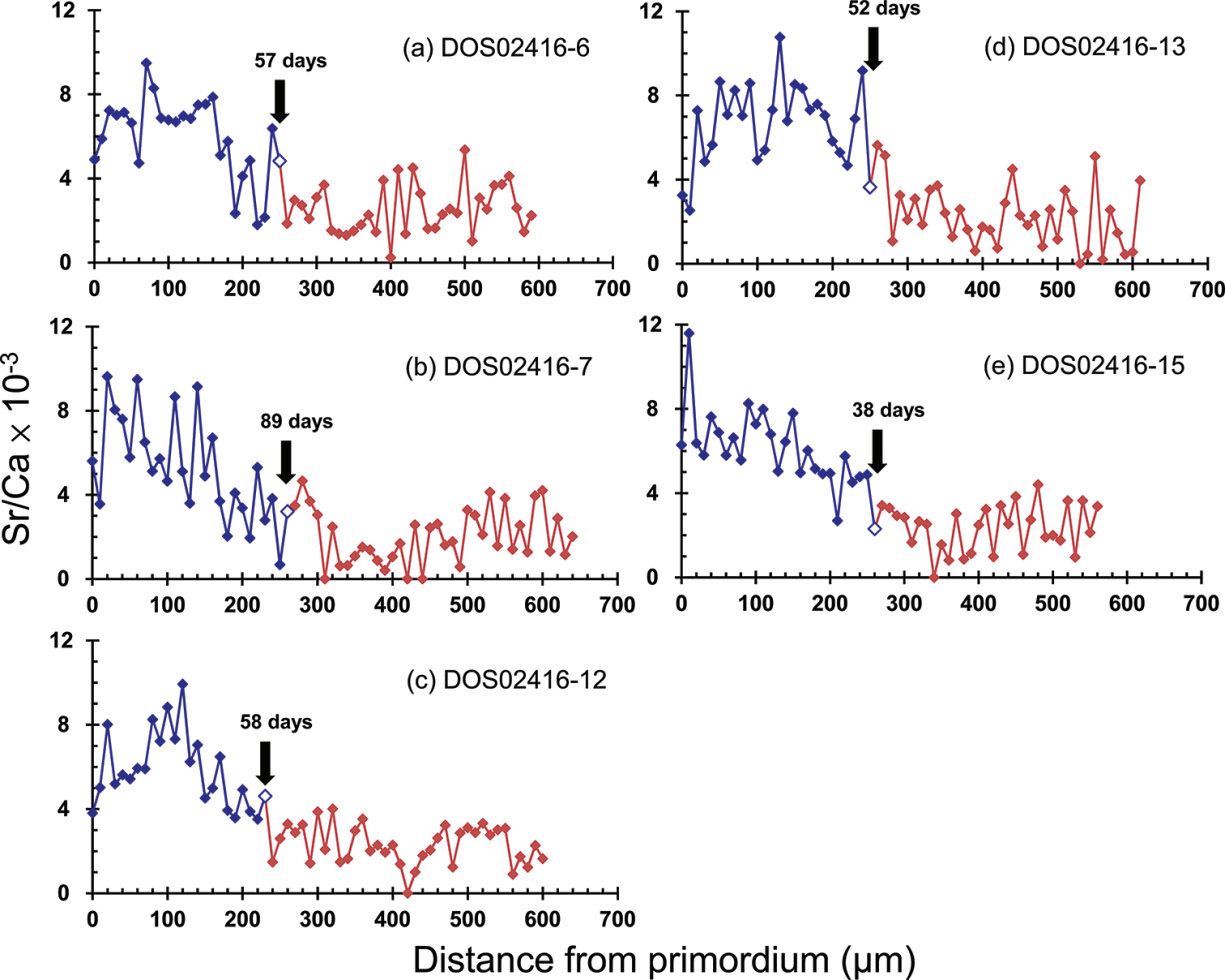
Analyzed transects of otolith Sr:Ca ratios from the core to the edge of sagittal otoliths of *Rhinogobius formosanus* collected in the Malian Creek of northern Taiwan. The arrows represent the formation days of the otolith check mark counted from the core. a-e represent their catalog numbers.

**Figure 5.**
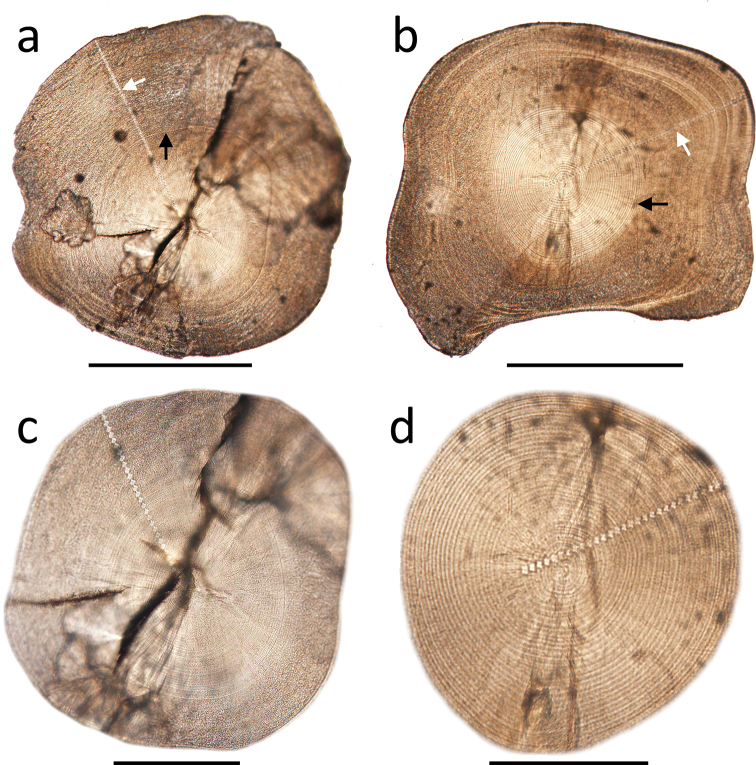
Otolith microstructure of an amphidromous goby (DOS02416-7, panel **a** and **c**) and **a** landlocked goby (DOS03534-19, panel **b** and **d**). Panel **c** and **d** illustrate the clear growth increments during the pelagic larval duration of the goby. The white arrows in panel **a** and **b** indicate the transect of otolith Sr:Ca ratio analysis. The black arrows indicate the otolith check mark where otolith Sr:Ca ratios drop from marine to freshwater signatures (panel **a**) and structural transition from clear concentric ring to ambiguous rings (panel **b**), respectively. Scale bars: 500 μm for panel **a, b**; 100 μm for panel **c, d**.”

For the gobies collected in MLC, five individuals showed high Sr:Ca ratios (approximately 5–10 × 10^-3^) from the otolith core to around 200 to 300 μm, followed by low otolith Sr:Ca ratios (approximately 0–5 × 10^-3^) to the edge (Fig. 4a–e). The high-to-low variations of otolith Sr:Ca ratios suggested that the fish had a planktonic stage in the sea followed by a freshwater residence as found in other species (e.g., Shen et al. 1998; Shiao et al. 2015). A high‐contrast growth increment, namely an otolith check, appeared at the transition of high-to-low otolith Sr:Ca ratios (Fig. 5a). The mean (± standard deviation) otolith Sr:Ca ratios before the otolith check varied between 5.1 ± 2.4 × 10^-3^ and 6.6 ± 2.0 × 10^-3^ among the fish, which were significantly larger than the values (2.0 ± 1.3 × 10^-3^ to 2.5 ± 1.2 × 10^-3^) beyond the otolith check (one-way ANOVA, F = 389.9, P < 0.01). It is likely that the otolith check was formed when the gobies migrated from the sea into the river during the post-larval or early juvenile stages as found in other amphidromous goby species (Shen and Tzeng 2002). Therefore, the growth increments before the otolith check were defined as the marine PLD, which varied from 38 to 89 rings with the mean value of 58.8 ± 18.7 rings (Table 2; *N* = 5).

**Table 2.** Otolith growth increments (days) and growth rate (μm d^-1^) corresponding to the pelagic larval duration of *Rhinogobius formosanus*. FR, Feitsui Reservoir, MLC, Malian Creek. SL for standard length in mm. na for data not available due to damage of specimens.

**Locality**	**Catalog number**	**SL**	**Otolith growth increments**	**Otolith growth rates**
FR	DOS03534-11	31.0	44	4.3
	DOS03534-14	na	46	3.9
	DOS03534-15	na	45	5.2
	DOS03534-16	32.6	36	6.3
	DOS03534-19	na	39	5.9
	DOS03534-33	na	24	7.4
	DOS03534-35	31.0	40	5.5
	DOS03534-37	31.5	36	5.3
	average ± SD	31.5 ± 0.8	38.8 ± 7.1	5.5 ± 1.1
MLC	DOS02416-6	31.3	57	4.8
	DOS02416-7	33.5	89	3.4
	DOS02416-12	29.4	58	5.6
	DOS02416-13	30.2	52	6.1
	DOS02416-15	na	38	6.7
	average ± SD	31.1 ± 1.8	58.8 ± 18.7	5.3 ± 1.3

**Figure 6.**
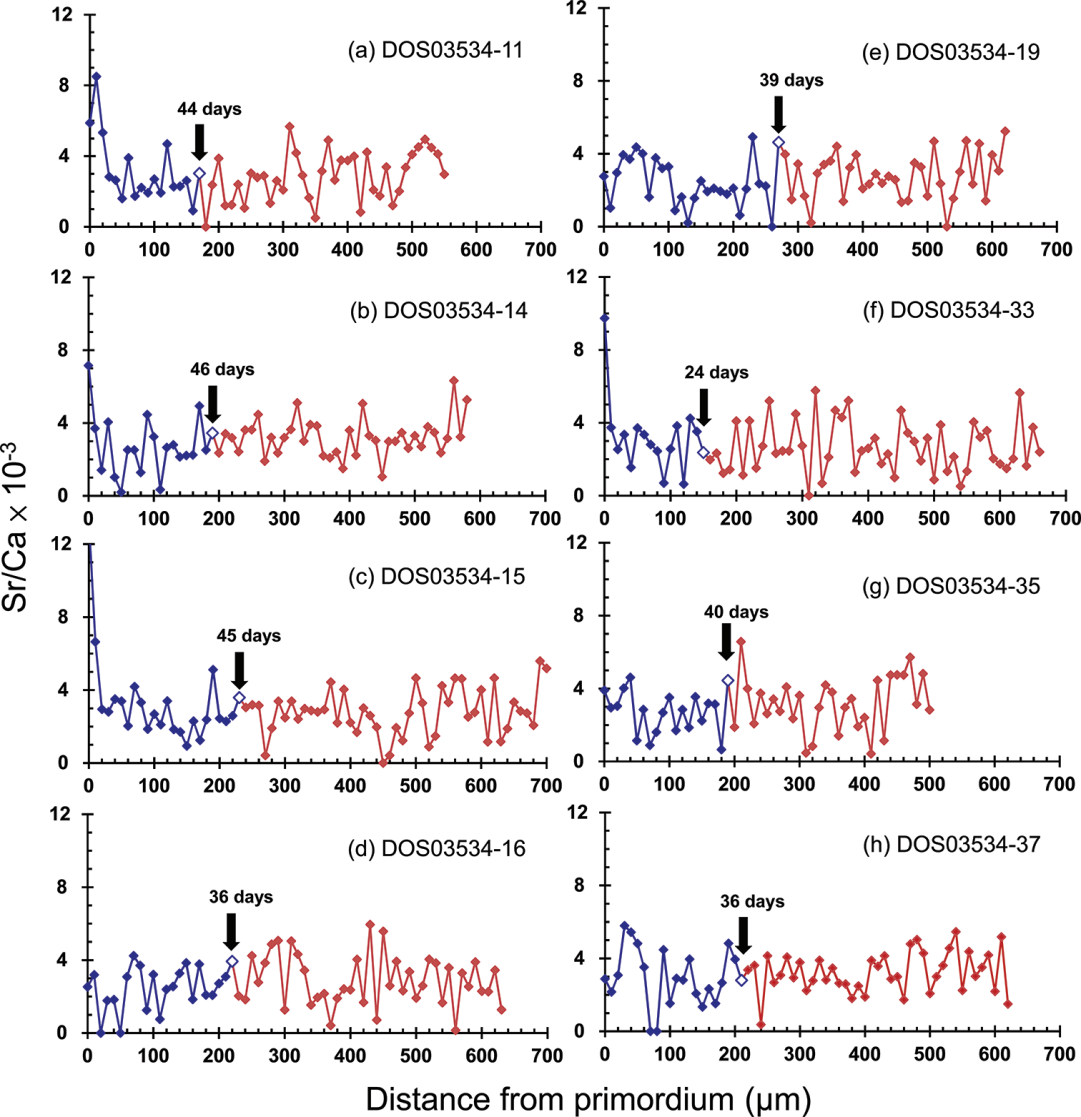
Sr:Ca ratios along transects from the core to the edge of sagittal otolith of *Rhinogobius formosanus* collected in the Feitsui Reservoir. a-h represent their catalog numbers.

A different pattern of consistently low Sr:Ca ratios from the otolith core to the edge was found in all the gobies collected in the Jingualiao Creek although some fish showed one or two relatively higher Sr:Ca ratios (Fig. 6). In addition, the high-contrast growth increment otolith check was not observed in the early life stage of these gobies (Fig. 5b). These results suggested that these gobies did not migrate to the sea and spent their whole life in the creeks. The gobies examined showed clear and concentric growth increments in the inner part of the otolith then the growth increments became inconspicuous in the outer area. The transition from clear to ambiguous growth increments were regarded as the end of PLD, as observed in many species (e.g., Victor 1986; Raventós and Macpherson 2001). The otolith growth increments from the core to the structural transition varied between 24 and 46 rings with a mean of 38.8 ± 7.1 rings. These results suggested that the landlocked gobies might have a PLD between 24 and 46 days (Table 2; *N* = 8), which was significantly shorter than the PLD of the amphidromous gobies (student’s t-test, t = 2.79, P = 0.018). However, the mean otolith growth rate before the settlement was similar between FR (5.5 ± 1.1 μm d^-1^) and MLC (5.3 ± 1.3 μm d^-1^) populations and not statistically significant (student’s t-test, t = 0.26, P = 0.80).

## Discussion

Molecular analyses show haplotypes of FR and MLC populations are mixed without reciprocal monophyly (Fig. 3), implying that gobies of these two populations are conspecific and the observed otolith differences can be considered intraspecific variations. Results of the present study support our first hypothesis that the population of *R. formosanus* in the creek discharging into the reservoir is landlocked based on the data of consistently low otolith Sr:Ca ratios throughout life (Fig. 6; Tsunagawa and Arai 2008), rather than artificially released founders with an amphidromous signature. Otolith Sr:Ca ratios were mainly related to the water Sr concentration or salinity (Tran et al. 2019). However, physiological and water temperature might also affect otolith Sr:Ca ratios (Elsdon and Gillanders 2002). Therefore, one or two analyzed spots with Sr:Ca ratios > 5 ×10^-3^ in the otoliths of FR goby were not regarded as a marine signal but might be an analytical artifact due to the microstructure defects such as cracks, an unsmooth otolith surface or being influenced by the organic composition (Goldstein et al. 1992; McFadden et al. 2015). This result is in line with expectations since the dam of the FR is too high to be ascended by the fish. Various kinds of dam constructions have become major impediments to freshwater fish for the upstream migration in the rivers of Taiwan (Chang et al. 1999). In contrast, the gobies collected in MLC were all amphidromous (Fig. 4). These results imply that *R. formosanus*, like other goby species namely, *Rhinogobius* spp., the cross-band type, the large-dark type, the dark type, the cobalt type, the orange type (Sakai et al. 2004; Tsunagawa and Arai 2008), is a facultatively amphidromous goby, which can develop normally, grow, and complete their life cycle in freshwater environments.

To the best of our knowledge, very little comparative data, if any, between the amphidromous and landlocked gobies has been reported. The present study found that the PLD of *R. formosanus* were on average 20 days longer in the amphidromous population compared with the landlocked population based on the assumption that the otolith growth increments were deposited in a daily cycle as found in many goby species (e.g., Hoareau et al. 2007; Maeda et al. 2007; Taillebois et al. 2012; Shiao et al. 2015). Our results are different from previous studies that found prolonged exposure to freshwater may postpone development of goby larvae (Lindstrom and Brown 1994; Yokoi and Hosoya 2005). Variations of PLD between populations have been found and the mechanisms examined in many fish species (Sponaugle and Cowen 1994; Sponaugle et al. 2002; Huang et al. 2018). The timing of larval metamorphosis may be either size-, age-, or habitat-dependent (Benoît and Pepin 1999; Shen and Tzeng 2008). In the case of amphidromous *R. formosanus*, there are large variations in PLD of 38–89 days while the landlocked *R. formosanus* has shorter and less variable PLD of 24–46 days. Therefore, the triggering of metamorphosis of this species is probably not age-dependent. The size of larvae, freshwater discharge and suitable benthic habitats may be the vital factors triggering metamorphosis. However, all our specimens are either adult or juvenile, and it is not possible to evaluate size at metamorphosis. Nevertheless, otolith growth was usually closely related to somatic growth (Campana and Neilson 1985). The otolith growth rate was very similar between the FR and MLC populations, suggesting a similar somatic growth rate of the larval gobies either in the sea or in the river discharging into the reservoir. This implies that size may not be a concern since gobies of FR and MLC undergo metamorphosis at different PLDs and therefore supposed to be of different sizes. The habitats or environments experienced by *R. formosanus* larvae may explain the different PLD between the amphidromous and landlocked populations. The longer PLD of amphidromous gobies is likely due to the time needed for hatched larvae to drift downstream from the creek to the sea, the feeding and growth to post-larvae in the sea, and the time for actively searching and swimming to the estuary (Keith 2003). Although amphidromous larval gobies tend to stay in coastal areas (Sorensen and Hobson 2005), larval transportation into the open ocean may occur in extreme situations. It is likely that the gobies experiencing longer dispersion will need more time to come back to the estuaries in the original or nearby areas. However, long dispersion may also lead to the death of the larval gobies if a suitable benthic habitat is not encountered when the maximal plasticity of the PLD is reached. Therefore, a longer and more variable PLD (38–89 days) is likely due to the complex amphidromous life history of *R. formosanus*. On the other hand, the stable environment in the creek or in the reservoir (Kolding and Zwieten 2012) may facilitate the larval development in a shorter and less variable time for the landlocked population. Furthermore, a shorter PLD can facilitate an earlier habitat change of larval fish from the upper water column to settlement in benthic habitats where shelter is more abundant. Therefore, a shorter PLD may enhance the survival rate of a pelagic larval goby living in a creek connecting to the reservoir.

Pelagic larval duration, usually considered a measure of dispersal potential, has been shown to be positively correlated to range size and negatively correlated to species richness, implying that PLD may regulate speciation rate as an evolutionary mechanism (Lester and Ruttenberg 2005). Selkoe and Toonen (2011) provide new insight at the molecular level and conclude that PLD is negatively correlated to isolation by distance, further supporting the concept of an evolutionary mechanism. *Rhinogobius formosanus* may have a PLD as long as three months as observed in this study, which may allow the larvae to disperse over hundreds of kilometers, depending on the current speed and ocean hydrodynamics, and explain the wide distribution of this species in the southeastern coast of China and the northern coast of Taiwan. Due to lack of genetic differentiation between FR and MLC populations, the variation in the PLD of *R. formosanus* is probably a consequence of acclimatization rather than adaptation.

Molecular analyses show haplotypes of FR and MLC populations are mixed without reciprocal monophyly and the genetic diversity of the former is much lower than the latter (Table 1). Lower genetic diversity might be a consequence of a founder effect and imply an artificial released population in FR (Tzeng et al. 2005; Hamner et al. 2007). On the other hand, a small native population isolated after the construction of the dam may also result in the same genetic pattern. However, the present data is not able to answer the origin of the FR population and range wide population genetic study on this species may be needed to provide more information.
